# Mitochondrial Function and Oxidative Stress Biomarkers in Diabetic Retinopathy Development: An Analytical Cross-Sectional Study

**DOI:** 10.3390/ijms252313084

**Published:** 2024-12-05

**Authors:** Ricardo Raúl Robles-Rivera, Fermín Paul Pacheco-Moisés, Cecilia Olvera-Montaño, José Alberto Castellanos-González, Andre Leonardo Barley-Villaseñor, Ernesto Germán Cardona-Muñoz, Adolfo Daniel Rodríguez-Carrizalez

**Affiliations:** 1 Institute of Clinical and Experimental Therapeutics, Department of Physiology, Health Sciences University Center, University of Guadalajara, Guadalajara 44340, Jalisco, Mexico; ricardo.robles3334@alumnos.udg.mx (R.R.R.-R.); cecilia.olvera3372@alumnos.udg.mx (C.O.-M.); andre.barley@hotmail.com (A.L.B.-V.); german.cardona@academicos.udg.mx (E.G.C.-M.); 2 Department of Chemistry, University Centre of Exact and Engineering Sciences, University of Guadalajara, Guadalajara 44430, Jalisco, Mexico; fermin.pacheco@academicos.udg.mx; 3 Department of Ophthalmology, Specialties Hospital of the National Occidental Medical Center, Mexican Institute of Social Security, Guadalajara 44349, Jalisco, Mexico; jose.castellanos2223@alumnos.udg.mx

**Keywords:** diabetic retinopathy, oxidative stress, mitochondrial function

## Abstract

DR is a complex complication of DM with multiple biochemical pathways implicated in its genesis and progression. Circulating OS and mitochondrial function biomarkers represent potential candidates in the DR staging system. We conducted a comparative cross-sectional study comparing the OS biomarkers: TAC, GR, NOS, CARB, and hydroperoxydes, as well as mitochondrial function biomarkers: ATP synthase and ATPase activity in healthy volunteers, DM w/o DR, Moderate and Severe NPDR, and PDR. TAC is progressively diminished the more DR progresses to its proliferative stages. GR and NOS may function as biomarkers to differentiate the progression from S NPDR to PDR. CARB may correlate with the progression from M NPDR to S NPDR. Hydroperoxide levels were higher in patients with DR compared to DM w/o DR expressing OS in the early development of DR. ATPase activity is increasingly augmented the more DR progresses and may function as a biomarker that reflects the difference between N PDR and PDR, and ATP synthesis was lower the more DR progressed, being significantly lower compared to DM w/o DR. The behavior of OS and mitochondrial function in several stages of DR may aid in the staging and the prognosis of DR.

## 1. Introduction

Currently, we are going through a global pandemic of diabetes mellitus (DM), where it is estimated that by 2030, 643 million people will be affected by this chronic disease. This number is also expected to rise to 783 million by 2045, about 10% of the global population [1]. Diabetic retinopathy (DR) is the most common complication of DM, being one of the main causes of blindness in the world among the working population [2], and it is expected to increase in prevalence by up to 55.6% by 2045 [3]. DR involves a complex interplay between multiple biochemical alterations, caused by changes in the chorioretinal neurovascular couple [4,5], causing clinical manifestations in the retina that acutely or chronically progress neovascularization, causing anything from visual impairment to blindness [6,7].

DR biogenesis and progression are associated with mitochondrial dysfunction, because the constant toxic exposure of high systemic levels of glucose due to insulin resistance affects tissue homeostasis, leading to retinal apoptosis and progressive damage to the retina [8,9,10]. Mitochondria are intracellular organelles playing the role of metabolizing nutrients and they are the main source of energy, responsible for various processes in the organism, such as energy metabolism, cell survival, and death [11,12,13]. Pathophysiological alteration in mitochondria has been seen in aging and metabolic disorders such as DM, altering mitochondrial morphology and function and impairing many processes such as the oxidative capacity and antioxidant defense [9,10,14,15,16]. The retina is a highly active tissue, with high demands of ATP and, thus, high mitochondrial activity, and DM-induced alterations of neuronal cells and photoreceptors may be associated with mitochondrial dysfunction, as shown by clinical and preclinical studies, supporting the hypothesis that visual impairment is preceded by neurodegeneration and vascular damage [17,18,19,20,21]. Mitochondrial function biomarkers, then, may have diagnostic and/or prognostic value in DR staging and progression.

Oxidative stress (OS) is defined as the state where oxidant agents outweigh the antioxidant defenses of a cell [22,23]. OS is highly linked to DR, as the production of Reactive Oxygen Species (ROS) acts as the common pathway to said biochemical alterations, for example, to inflammation, nitrosative stress, endoplasmic reticulum stress, neurodegeneration, DNA damage, and mitochondrial dysfunction [8,11,24,25]. Some sources of ROS include NADPH oxidases, the mitochondrial electron transport chain, nitric oxide, cytochrome P-450, and cyclooxygenase. Furthermore, there is cross-talk involved in mitochondria-induced angiogenesis, focusing on Vascular Endothelial Growth Factor (VEGF) signaling where the oxidative state produced by a dysfunctional mitochondria amplifies excess ROS, impairing the homeostatic maintenance of the vasculature and inducing angiogenesis, characteristic of Proliferative Diabetic Retinopathy (PDR) [5,23,26,27,28,29].

While DR is a disease for which treatment options are only available after complications have occurred, such as vitreous hemorrhage, neovascularization, diabetic macular oedema, or retinal detachment [30], the surge of biomarkers in favor of the prediction, diagnosis, and prevention of DR are needed [31,32,33,34,35,36]. Taking this into account, the present study aims to extend current knowledge on molecular and biochemical biomarkers of OS and mitochondrial function and their role in the clinical manifestation of real-life patients with DR at two different stages of development, before they present angiogenesis, and in patients with PDR. This behavior will be compared to patients who have not developed this microvascular complication but have a DM diagnosis, as well to healthy volunteers.

## 2. Results

### 2.1. Clinical Parameters

A total of 32 subjects were included in this study, 18 men aged 61.5 ± 8.05 and women aged 64.21 ± 7.38. The population selected was divided as follows: 12 patients had a Moderate NPDR status, 11 had a Severe NPDR status, and, lastly, 9 patients already had neovascularization and thus were classified as PDR. The demographic data of the participants are shown in Table 1, where, for comparison purposes, values corresponding to DM w/o DR patients were added.

Using the Kruskal–Wallis test with post hoc correction with the Dunn–Bunferroni test, our findings show a difference in proportions of the male to female ratio between groups (*p* = 0.020); however, when comparing each group individually, this is only statistically significant when comparing the M NPDR to the PDR (3:9 male to female ratio compared to 8:1, *p* = 0.021). Other than that, there are no other differences when comparing the groups that have already presented DR, only when compared to patients without the microvascular complication. When comparing all groups with each other, our population had a statistically different age mean, prevalence of hypertension, and years of DM duration and DR duration (*p* = < 0.001, 0.007, and <0.001 and <0.001, respectively); however, this difference was due to DM w/o DR group values, since we found no statistical difference when comparing groups who already have DR.

The clinical and ophthalmological parameters can be found in Table 2. Weight by itself and Body Mass Index (BMI) were statistically significant when comparing within groups (*p* = 0.049 and <0.001, respectively), but no statistically significant difference was found when comparing individually between DR groups. Systolic Blood Pressure (SBP) was statistically higher in PDR when compared to S NPDR, but not when compared to M NPDR (*p* = 0.033 and *p* = 0.116, respectively). There was a statistically significant difference in Diastolic Blood Pressure (DBP) between groups, but this was not statistically significant when comparing between patients who had already developed DR. When comparing visual acuity, the Best Corrected Visual Acuity (BCVA) of patients was different statistically speaking (*p* = < 0.001 and *p* = 0.006 for the Right Eye (OD) and the Left Eye (OS), respectively), within groups. Further exploration within DR groups showed that PDR BCVA was significantly lower when compared to both M NPDR (*p* = 0.005 for the OD, and *p* = 0.006 for the OS) and S NPDR (*p* = 0.024 for the OD, and *p* = 0.032 for the OS). There was no difference regarding intraocular pressure (IOP), weight or BMI in the three groups.

To evaluate the confounding parameters, laboratory tests regarding metabolic, kidney health, and liver health parameters can be found in Table 3. Regarding glucose regulation, an expected upwards trend can be seen as the DR progresses, as expected from the natural evolution of the disease (with a statistically significant difference when comparing groups of *p* = 0.001 for both HbA1c and fasting glycemia); nevertheless, only when comparing M NPDR to PDR was this increase statistically significant (*p* = 0.005). When reviewing kidney function between study groups, Creatinine (Cr) was significantly different between groups (*p* = 0.011). Where Cr was higher when comparing M NPDR to PDR (*p* = 0.004), the estimated Glomerular Filtration Rate (eGFR) was not different between DR groups, but a statistically significant difference was found when comparing all four groups (*p* = 0.002). Regarding liver function, when comparing Aspartate transaminase (AST) levels between groups, a statistically significant difference was found (*p* = 0.001), and paradoxically, M NPDR had higher levels compared to PDR (*p* = 0.036), but these levels are within the clinically healthy values. Similarly, Alanine transaminase (ALT) levels were significantly different between groups (*p* = 0.004); however, there was no difference when comparing DR groups individually. Lastly, regarding lipid profile, Total Cholesterol (TC) was different when comparing all groups with each other (*p* = < 0.001). A trend could be seen where the more advanced DR was, the higher the TC levels were (122.1 ± 25.35 for DM w/o DR, 159.09 ± 20.02 for M NPDR, 170.36 ± 18.6 for S NPDR, and 198.22 ± 25.62 for PDR), and this was statistically significant when comparing M NPDR to PDR (*p* = 0.001) and S NPDR to PDR (*p* = 0.025), but not when comparing M NPDR to S NPDR (*p* = 0.094). When comparing HDL levels within groups, these were significantly lower when comparing M NPDR to PDR (*p* = 0.035); however, LDL levels tended to be higher the more advanced DR was, but not to a statistically significant extent.

### 2.2. Oxidative Stress and Mitochondrial Function Biomarkers

OS and mitochondrial function biomarkers can be seen in Table 4. OS status can be evaluated globally with Total Antioxidant Capacity (TAC), where the more DR progresses, the less TAC mEq/mL, where PDR group showed significantly lower TAC than M NPDR (*p* = 0.033) and S NPDR (*p* = 0.053). Regarding Glutathione Reductase (GR) there was only a significant decrease when comparing S NPDR to PDR (*p* = 0.024). There was an upwards trend regarding Nitric Oxide Synthase (ONS), where from DM w/o DR patients to S NPDR patients, the ONS levels were increasingly higher; however, in PDR patients the levels would be notably depleted, without being statistically significant. The oxidant biomarker carbonyl groups in proteins (CARB) had a similar trend, where the more severe the microvascular complication was, the higher the CARB levels. However, only when comparing M NPDR to S NPDR was this statistically significant (*p* = 0.034). Finally, regarding H_2_O_2_, this showed a similar trend to ONS, where there was an upwards trend peaking at M NPDR, and when progressing, levels tended to decrease without showing a significant difference when comparing groups.

Mitochondrial function was indirectly assessed with ATPase and synthesis. ATPase activity significantly increased, especially in PDR groups compared to M NPDR and S NPDR (*p* = 0.003 and *p* = 0.002, respectively). On the other hand, ATP synthesis showed a trend where the further DR progressed, the more synthesis was diminished, without statistical significance.

**Figure 1:**
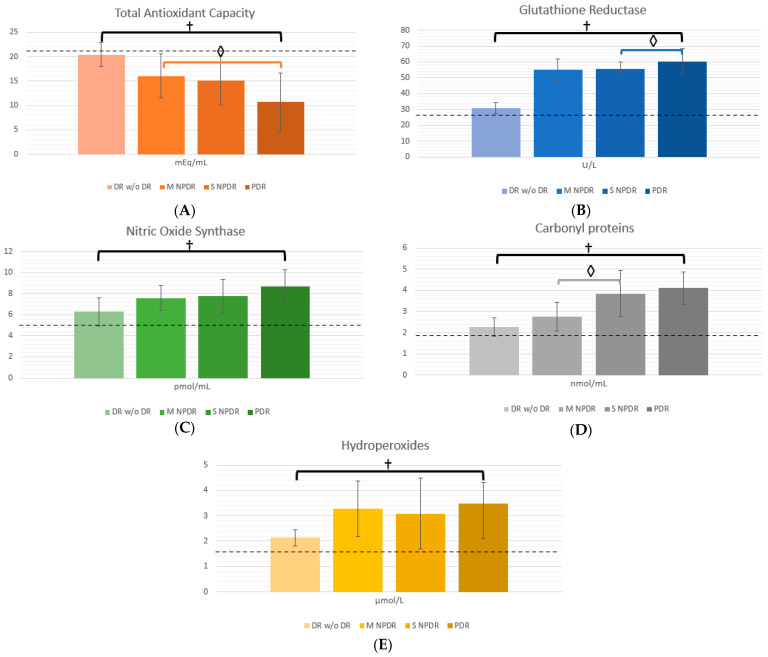
Oxidative Stress biomarkers in patients with Diabetic Retinopathy. (**A**) shows Total Antioxidant Capacity expressed as mEq/mL; (**B**) Glutathione Reductase levels expressed as U/L; (**C**) Nitric Oxide Synthase expressed as pmol/mL; (**D**) Carbonyl groups in proteins expressed as mmol/mL; and (**E**) Hydroperoxides expressed as µmol/L. Columns are expressed with mean and error bars representing standard deviation. For reference, dotted line represents the mean value for healthy volunteers. ◊ = statistical significance, † = statistical significance between all groups. KW = Kruskal–Wallis with post hoc Dunn–Bonferroni test was used to compare differences between all groups. Mann–Whitney U-test was used to compare differences between DR groups (M NPDR vs. S NPDR, M NPDR vs. PDR, and S NPDR vs. PDR).

**Figure 2:**
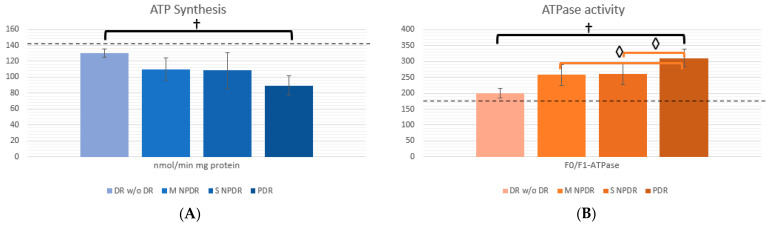
Mitochondrial function biomarkers in patients with Diabetic Retinopathy. (**A**) shows ATP synthesis expressed as nmol/min mg protein; (**B**) ATPase activity is expressed as F0/F1-ATPase. Columns are expressed with means and error bars represent standard deviation. For reference, the dotted lines represent the mean value for healthy volunteers. ◊ = statistical significance between selected DR groups, † = statistical significance between all groups.

To illustrate biomarker behavior, Figure 1A–E show a column chart for each OS biomarker, and Figure 2A,B show mitochondrial function biomarkers. In each chart, a dotted line can be seen, which represents the levels expressed by healthy volunteers for each biomarker (levels for healthy volunteers were as follows: TAC: 22.15 ± 2.16 mEq/mL; GR: 27.11 ± 27.11 ± 3.16 U/L; NOS: 4.92 ± 0.31 pmol/mL; CARB: 1.91 ± 0.154 nmol/mL; H_2_O_2_: 1.63 ± 0.152 µM/L; ATPase: 175.2 ± 6.704 F0/F1-ATPasa; and ATP synthesis: 142.96 ± 7.841 nmol/min mg protein). The graphs illustrate the mean and standard deviation for each biomarker per group.

As expected, TAC representing a form of global antioxidant power was notably higher in patients without DM, and the trend is visually represented in Figure 1A, where the more the DR has progressed, the lower the TAC levels. In Figure 1B–E, on the other hand, pro-oxidant biomarkers are shown, and thus, lower levels can be seen in healthy volunteers compared to patients who have already developed DM, and DR.

On the one hand, the mitochondrial function biomarkers in Figure 2 show that ATP synthesis is markedly increased in healthy volunteers, whereas on the other hand, the activity of ATPase is lower in healthy volunteers compared to patients with DM w/o DR, and patients with DR.

## 3. Discussion

The present article is a cross-sectional analytic study that aims to increase the understanding of the behavior of mitochondrial and OS in a real-life setting of patients with DR.

Currently, the widely utilized approaches to treat DR include pan-retinal photocoagulation therapy, anti-VEGF corticosteroid therapy, or surgical management, but these are mainly reserved for advanced cases. Novel candidate medications and approaches are aimed at newer molecular targets in the pathogenesis of DR specifically designed for each patient or for earlier stages of DR, preventing or slowing down its progression [37,38,39,40], and thus expanding the knowledge on biomarker behavior in a healthy volunteer during the progression though several stages of DR is crucial.

### 3.1. Clinical and Ophthalmological Parameters

Several risk factors have been associated with the development and progression of DR, the main risk factor being a constant and prolonged lack of glycemic control [37,41,42], and that is no different in our findings. We found an upwards trend regarding HbA1c between groups. As reported previously, HbA1c control directly correlates with the progression of DR, and thus represents an important variable that may impact OS and mitochondrial function markers. This cannot be said for fasting glucose levels, as there is no clear trend between groups. Lipid metabolism also plays a role in the development of microvascular complications [43]. Our findings showed that the further DR progressed, the higher the TC levels presented in each group, and a statistically significant difference was found when comparing all groups with each other (*p* = < 0.001); however, no difference was found when comparing DR groups among themselves. Lipid metabolism influences DR progression. However, since the subcategorized patients with DR did not have a statistical difference between each other, this is not a confounding variable for biomarker levels. In a similar manner, a markedly diminished BCVA was found in patients on PDR stage. This finding was expected as the natural progression of the disease, and correlates with Lupión–Durán et al.’s [44] study, which evaluates other markers of visual function in different stages of DR. Importantly, serum OS or mitochondrial function biomarkers may offer diagnostic or prognostic value, preventing visual impairment or DR progression in the future.

Other potentially confounding variables that were found to be different between groups include age distribution (*p* = < 0.001), blood pressure for both Systolic Blood Pressure (SBP) (*p* = < 0.001) and Diastolic Blood Pressure (DBP) (*p* = < 0.001), and the years with a diagnosis of DM (*p* = < 0.001). Regarding age distribution and DM diagnosis duration, no statistically significant difference was found when comparing DR groups with each other, meaning that the difference was associated with the DM w/o DR groups and the natural progression of the disease. When comparing DR groups among themselves, SBP was significantly higher in PDR statistically speaking, compared to M NPDR groups (*p* = 0.033), whereas there was no statistically significant difference when comparing DBP between DR groups. Even though hypertension as a comorbidity is correlated with the development of DM microvascular complications [37,45], the blood pressure values within DR groups are still within the normal to healthy values, and thus are not a confounding variable in our study. Cr, eGFR, Urea, and UA as kidney function variables were statistically different when comparing all groups (*p* = 0.011 for Cr, *p* = 0.002 for eGFR, *p* = < 0.001 for Urea, and *p* = 0.003 for UA). Cr was significantly higher in PDR compared to M NPDR (*p* = 0.004); however, both groups have a Cr value within the normal ranges and present no clinically significant difference. Similarly, UA was significantly higher in PDR compared to M NPDR groups (*p* = 0.022); however, this difference was not clinically significant, and no patients with a gout diagnosis were included. There was no statistically significant difference when comparing eGFR and Urea within DR groups. Regarding hepatic enzymes, AST levels were statistically different when comparing all groups (*p* = 0.001) and, paradoxically, AST is statistically higher in M NPDR than in S NPDR (*p* = 0.036). However, these liver enzyme levels are still within the healthy values, meaning that this may not be clinically significant. ALT levels were statistically different when comparing all groups (*p* = 0.004), but no significant difference was found when comparing DR groups. Even though NAFLD has been associated with diabetic microvascular complications [46], patients with a diagnosis were excluded.

It should be noted that the more risk factors, the greater the risk of developing DR [47], and, on the other hand, adhering to an overall healthy lifestyle, improving glycemic control, liver function, and lipid profile, is associated with a lower risk of microvascular complications of DM [48]. Therefore, taking all these clinical parameters into account as potentially confounding variables is relevant when discussing the progression of DR.

### 3.2. Oxidative Stress Biomarkers

OS has been an important therapeutic target for DR genesis and progression [5,19,27]. Currently, the progress in understanding early DR has aimed its efforts at identifying different “phenotypes” of DR, with the aid of biomarkers for personalized management [49,50]. Our findings suggest different behaviors of OS biomarkers depending on the stage of DR development.

#### 3.2.1. Antioxidant Biomarkers

Taking into account that the body has a physiological ability to neutralize oxidative imbalances, TAC acts as a biomarker that globally takes into account the antioxidant defense that enzymes and both large and small molecules may offer [51]. Our findings expressed a constant diminishing of TAC with the advancement of DR, with a statistically significant difference when comparing all groups (*p* = < 0.001), and this difference was significant when comparing M NPDR to PDR (*p* = 0.033), similarly to what was described by Andrés-Blasco, I. et al. [52]. This can be explained by how oxidant status overcame antioxidant defenses the more DR progressed, and would further explain our team’s previous findings [53], suggesting a negative correlation between the TAC and DR stages.

Other than TAC, GR is an enzyme that is partly responsible for modulation and maintaining redox homeostasis, by maintaining a supply of reduced glutathione [54,55]. Contrary to previous findings among Indian population [56,57], our findings suggest that GR levels tend to rise the more advanced DR is before the PDR stage, with a statistically significant difference between groups (*p* = < 0.001), and significantly lower levels when comparing S NPDR to PDR (*p* = 0.024). This finding suggests that GR in our patients was an enzyme that increased due to exposure to OS, trying to defend itself systemically from oxidant agents, but that this antioxidant mechanism is depleted when PDR is present. The quantification of reduced glutathione would reflect if the product of this enzyme was correlated with its activity, or if glutathione reductase was to be diminished despite its reductase activity.

#### 3.2.2. Oxidant Biomarkers: OS in DR Can Be Evaluated Though Several Approaches and Metabolic Pathways, Affecting Lipids and Cellular Membranes, Vasculature, and Proteins, to Name a Few

During the course of DM, nitric oxide (NO), a free radical, is generated from L-arginine by NOS, following hyperglycemia-induced OS [58,59]. The higher plasma levels of NO in patients with DR, compared to healthy individuals, is the result of high glucose levels, enhancing the production of NO due to up-regulated endothelial NOS expression. Also, the reaction between NO and superoxide anions (increased by ischemia and hypoxia) produces peroxynitrite, a toxic oxidant, affecting endothelial function [60,61,62,63]. Our findings suggest that NOS activity is increased the more DR progresses (Patients with Diabetes Mellitus without Retinopathy (DM w/o DR): 6.28 ± 1.34, M NPDR: 7.57 ± 1.18, S NPDR: 7.74 ± 1.62, and PDR: 8.6 ± 0.52), with this difference being statistically significant when comparing all groups (*p* = 0.002), and no significant difference within DR groups. Previously, it has been shown that in the early stages of type two DM, NOS expression is increased as a compensation mechanism, whereas advanced stages show diminished NOS, associated with endothelial dysfunction [64,65]. The fact that our findings suggest that there is increased activity of NOS in PDR may suggest that when neovascularization is present, NOS production paradoxically tries to compensate for endothelial damage; however, in this pro-oxidative state, the subsequent production of NO aggravates OS. This would align with Farkas, K. et al. [66], which show that even when healthy volunteers show lower levels of NO, the overproduction of NO in the event of a microvascular complication may paradoxically be toxic to the tissue.

Carbonyl groups can be introduced into proteins due to reactive carbonyl species, as a consequence of the oxidation of lipids or carbohydrates, and they can be seen as precursors of advanced glycation end products (AGEs), which play an important role in the pathophysiology of DM’s complications [67,68,69]. Our findings suggest that CARB are different when comparing all study groups (*p* = < 0.001), and furthermore, that they are higher in S NPDR compared to M NPDR (*p* = 0.034). Previously, Konukoğlu, D. et al. showed a similar behavior of CARB content in different stages of patients with angiopathy [70], while Cakatay, U. et al. [71] showed that protein carbonyls increased in patients with poor glycemic control, which is consistent with our findings. Now, we go even further, suggesting that CARB may function as sensitive early biomarkers that may differentiate the progression between M NPDR and S NPDR.

Under physiological conditions, the body produces ROS continuously as a by-product of cellular metabolism; however, when produced in excess, or when cleared poorly due to diminished antioxidant defenses, ROS cause harm to cellular lipids, proteins, and DNA production [72]. In DM, an altered polyol pathway due to high glucose levels causes a reductive imbalance, generating ROS such as H_2_O_2_, forming the first step in OS-induced damage and mitochondrial damage [5,26,73]. Our study suggests increased H_2_O_2_ levels when comparing DM w/o DR with those who have already developed DR (*p* = < 0.001), as there is no statistically significant difference when comparing DR groups to each other. To our knowledge, the present study is the first to compare H_2_O_2_ levels between several stages of the development of DR, showing its potential as an early biomarker before the onset of microvascular complications.

### 3.3. Mitochondrial Function Biomarkers

The mitochondria as organelles have multiple fundamental roles in the cell, including in the energy transformation process and the production and consumption of ROS. Thus, when dysfunction exists, a vicious cycle of harmful ROS exacerbates mitochondrial damage [16]. When assessing mitochondrial functionality in DR, multiple blood biomarkers were assessed to better estimate mitochondrial damage, given that lower ATP synthesis may indicate an attempt to reduce mitochondrial OS, whereas ATPase is the catabolic process with which to produce energy with ADP and inorganic phosphates [9,11,16,74,75,76,77,78].

In our study, patients with DM have lower activity levels of ATP synthesis compared to healthy volunteers (*p* = < 0.001), as there is no statistically significant difference when comparing DR groups. Other studies have shown indirectly that ATP synthesis is lower in patients with DM compared to patients without a DM diagnosis [79]. On the contrary, a significant difference was found in ATPase activity when comparing all groups (*p* = < 0.001). Moreover, ATPase activity was greatly increased in PDR group compared to NPDR stages (*p* = 0.003 when comparing M NPDR to PDR, and *p* = 0.002 when comparing S NPDR to PDR), indicating that when neovascularization is present, a high catabolic state can be found in PDR patients’ plasma, which is supported by previous similar preclinical findings [80,81]. More clinical studies need to be conducted to assess mitochondrial function biomarkers’ potential in DR.

## 4. Materials and Methods

An analytical cross-sectional study was carried out on subjects who were recruited from March 2018 to December 2020. The study adheres to the strengthening the reporting of observational studies in epidemiology (STROBE) statement: cross-sectional studies. This cross-sectional study was approved by the ethics committee of the National Committee of Scientific Research of the Mexican Institute of Social Security with the register number R-2018-785-110 and conducted in accordance with the principles of the Declaration of Helsinki. Written informed consent was obtained from all participants.

### 4.1. Study Population

One retina specialist in the Department of Ophthalmology at the Center of Specialties of the Mexican Institute of Social Security in Guadalajara, Jalisco, Mexico, performed the ophthalmic examination. The evaluation of DR was according to the diagnostic criteria of the American Academy of Ophthalmology 2001 Annual Meeting guidelines, by retinal examination using an Ophthalmoscope. The severity of Diabetic Retinopathy was according to the international clinical diabetic retinopathy (ICDR) severity scale criteria [82]. The study subjects were further stratified either into Non-Proliferative Diabetic Retinopathy (NPDR) or PDR. NPDR patients were subclassified further into Moderate (M NPDR) or Severe (S NPDR) status according to the ICDR criteria.

**Figure 3:**
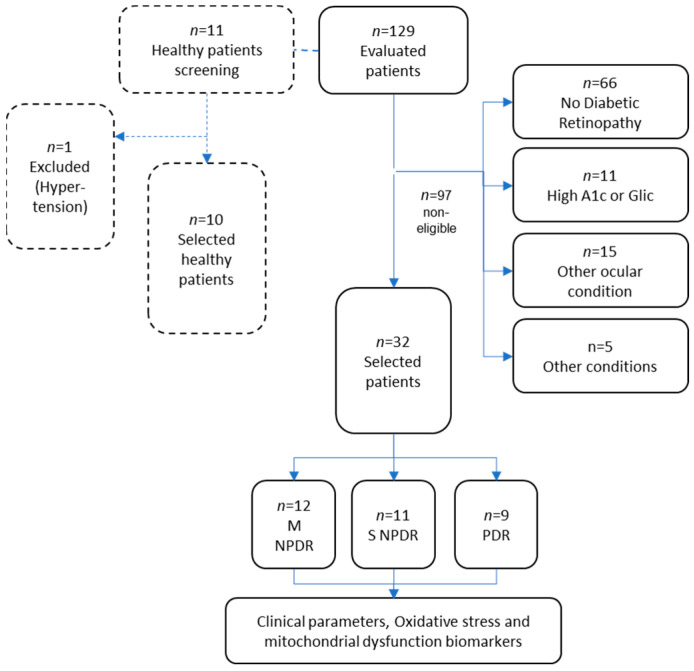
Flow diagram of the study. Other ocular conditions include glaucoma, macular oedema, cataract, corneal dystrophy, and/or macular degeneration. Other conditions include a history of or active smoking or alcohol abuse, and endocrine or autoimmune conditions. Abbreviations: A1c = Glycated hemoglobin; Glic = fasting glucose levels; DM w/o DR = diabetes mellitus without Diabetic Retinopathy; M NPDR = Moderate Non-Proliferative Diabetic Retinopathy; S NPDR = Severe Non-Proliferative Diabetic Retinopathy; and PDR = Proliferative Diabetic Retinopathy.

A total of 129 patients were evaluated, where 97 were not eligible, breakdown can be seen in the flow diagram of the study presented in Figure 3. Out of the 32 selected patients, 20 were stratified into NPDR and 12 into PDR. As a control group, 10 patients with DM and without DR (DM w/o DR) were selected to participate, and these patients were evaluated by the study team’s ophthalmologist to rule out any retinal damage. Other chronic, acute, or autoimmune conditions that may alter OS and mitochondrial function biomarkers were ruled out. Ten healthy volunteers were included to strengthen discussion of findings. The selection criteria for the population are as follows:

Inclusion criteria

- Signed informed consent;
- 18–75 years of age;
- Men or women with Moderate or Severe NPDR, without clinically significant macular edema;
- Men or women with PDR, without clinically significant macular edema.

Exclusion criteria

- Consumption of antioxidant supplements within 6 months of prior enrollment;
- History of severe cardiovascular disease;
- History of blood dyscrasias;
- History of cancer or neoplasia;
- History of liver or kidney disease;
- Pregnancy or lactation;
- Other ocular disease (glaucoma, cataract, corneal dystrophy, or macular degeneration);
- Participation in another clinical trial.

### 4.2. Sample Size Calculation

Sample size was calculated using the following formula $n=\frac{Z^{2}P\left(1-P\right)}{d^{2}}$ [83], where *n* was calculated using a 95% confidence interval and the expected prevalence was estimated using Konukoğlu, E. et al.’s [70] study with a precision of 0.17, with an *n* value of 10.33.

### 4.3. Data Collection

Baseline characteristics, demographical, habits, and other health-related information for each participant were obtained by standardized face-to-face questionnaires (validated by the authority) performed by the staff of the institutes during the cross-sectional study, and are as follows:

#### 4.3.1. Basic Information

Age, date of birth, diabetes duration, Diabetic Retinopathy duration, and history of hypertension and/or dyslipidemia. Medical history regarding medications used and/or other comorbidities was also recorded.

#### 4.3.2. Physical and Ophthalmological Examination

On all participants a generalized physical exam was conducted. The ophthalmological examination included evaluation and documentation of the anterior and posterior segment, measurement of Best Corrected Visual Acuity (BCVA), and intraocular pressure (IOP).

### 4.4. Laboratory Tests

Sample handling: ten milliliters of peripheral venous blood was collected and plasma and blood cells were separated.

#### 4.4.1. Biochemical Parameters

To assess confounding clinical parameters, all patients had the following tests performed:

- Metabolic parameters: Glycated hemoglobin (HbA1c), fasting blood glucose, Total Cholesterol (CT), High-Density Lipoprotein Cholesterol (HDL), Low-Density Lipoprotein Cholesterol (LDL), and Triglycerides.
- Kidney health: Creatinine (Cr), Urea, Uric Acid, and estimated Glomerular Filtration Rate (eGFR).
- Liver health: Aspartate transaminase (AST), Alanine transaminase (ALT), and Total Bilirubin (TB).

#### 4.4.2. Mitochondrial Function Biomarkers Methodology

Isolation of submitochondrial particles in platelets: 1 mL of plasma was centrifuged for 15 min at 5600 rpm at 4 °C. The resulting pellet was resuspended with platelet buffer and stored at −80 °C until processing.

ATPase activity: The reaction medium for ATPase activity contained 125 mM of KCl, 40 mM Hepes (pH of 8), 2 mM of MgCl_2_, and 30 µL of the sample. The reaction was initiated by adding 20 µL of ATP 100 mM. After 10 min, the reaction was stopped by adding 200 µL of trichloroacetic acid (30%). Protein was removed by centrifugation at 5000× *g* for 5 min. To 800 µL of the supernatant we added 0.4 mL of 3.3% ammonium molybdate and 200 µL of 10% ferrous sulphate. Absorbance was read at 660 nm. The absorbance values were referred to a phosphate curve. Appropriate blanks with samples and ATP were performed.

ATP synthesis: The assay for ATP synthesis was carried out in platelets with a coupled enzymatic reaction. The reaction mixture (0.5 mL) contained the following: 46 units of hexokinase, 1 mM NADH, 20 mM of glucose, 0.1 mM EGTA, 2 mM inorganic phosphate, 2 mM MgCl_2_, 2 mM ADP, 40 mM Hepes (pH 7.5), and 125 mM of KCl [84]. After incubating the mixture at 37 °C for 2 min under shaking, samples were added. The reaction was stopped after 10 min by adding 50 µL of the stopping mixture (25 mM EDTA, 2 µM carbonyl cyanide m-chlorophenyl hydrazone, and 30 µg oligomycin). The reaction was boiled for 10 min and centrifuged at 5000× *g* for 5 min. After cooling, the samples were incubated for 10 min at 30 °C in the presence of 0.5 mM NADP and 30 units of glucose-6-phophate dehydrogenase. The absorbance of the samples was measured at 340 nm.

#### 4.4.3. Oxidative Stress Biomarkers

Carbonyl groups in proteins: To 200 µL of plasma samples we added 500 µL of 10 mM 2,4-dinitrophenylhydrazine in 2M of HCl, and incubated the mixture for 1 h at room temperature. Afterwards, 200 µL of trichloroacetic acid (30%) was added and samples were centrifuged at 14,000× *g* for 20 min. The resulting pellets were washed three times with 1 mL of ethyl acetate-ethanol (1:1, *v*/*v*). Then, 600 µL of 6 M guanidine hydrochloride was added to the pellets and incubated for 15 min at room temperature. Absorbance was measured at 370 nm.

Nitric oxide synthase: The activity of nitric oxide synthase was determined by the diazotization of sulfanilic acid by nitric oxide at an acidic pH and the subsequent formation of a complex with N-(1-naphtanyl) ethylenediamine, according to the method proposed by Durak et al. [85]. Briefly, plasma samples were diluted 1:1 with a buffer containing 40 mM Tris-HCl (pH of 7.5), 2 mM EDTA, 0.5 mM phenylmethylsulfonyl fluoride, 10 µg/µL leupeptin, and aprotinin. The reaction mixture contained 0.2 mL of diluted samples, 0.2 M arginine, and 1 mM NADPH, and was incubated at 37 °C for one hour. Next, 0.2 mL of 10 mM HCl, 100 mM sulfanic acid, and 60 mM N-(1-naphtyl) ethylenediamine were added. The absorbance of the samples was read at 540 nm.

Glutathione reductase: The glutathione reductase activity was determined by following the oxidation of NADPH to NADP+ during the reduction of oxidized glutathione at 340 nm. The reaction medium contained 0.1 M of potassium phosphate buffer (pH 7.0), 1 mM EDTA, 1 mM oxidized glutathione, and 0.1 mM NADPH. Controls without samples or oxidized glutathione were performed [42].

Hydroperoxides: To 100 μL of samples we added 900 μL of FOX solution (100 µM xylenol orange, 250 µM ammonium ferrous sulfate, 100 mM Sorbitol, and 25 mM H_2_SO_4_). Then, samples were incubated at room temperature for 30 min using the following recipe: 50 µL of the sample was added to 950 µL FOX-1 reagent, vortexed, and incubated at room temperature for 30 min. Flocculated material is removed by centrifugation. Samples were centrifuged at 1000× *g* for 10 min and absorbance was read at 560 nm and compared against a hydroperoxide (H_2_O_2_) standard [86].

Total Antioxidant Capacity: To determine the Total Antioxidant Capacity (TAC), expressed as equivalents of uric acid, a commercial colorimetric kit was used (Total Antioxidant Power Kit, No. TA02.090130, Oxford Biomedical Research, Rochester Hills, MI, USA) following the manufacturer’s instructions. The samples were diluted 1:40 with the dilution buffer provided. To 200 µL of samples we added 50 µL of copper solution and incubated them at room temperature for 3 min. Then, 50 µL of stop solution was added and absorbance was read at 450 nm.

### 4.5. Statistical Analysis

The Shapiro–Wilks and Mann–Whitney U tests were used to analyze non-parametric data. The biomarkers are shown as means ± standard errors. The Kruskal–Wallis test (KW) with the post hoc Dunn–Bonferroni test was used to compare the differences between the four groups (DM w/o DR, M NPDR, S NPDR, and PDR) for continuous variables. The Mann–Whitney U test was used to compare differences between DR groups (M NPDR vs. S NPDR, M NPDR vs. PDR, and S NPDR vs. PDR). Qualitative variables were expressed as frequencies and percentages, and the χ^2^ test was used. A *p* value of ≤0.05 was considered statistically significant, a power calculation was 80%, and the confidence interval was 95%; Zα = 1.96, alpha value = 0.05, Zβ = 0.84, and beta value = 0.10. All data were computed using an Excel database and SPSS PC for Windows version 21 (Chicago, IL, USA) was used for the statistical analysis. A complete case analysis approach was used to handle missing data.

## 5. Conclusions

DR has a complex pathophysiology where multiple pathways interplay in its genesis and progression. To evaluate the potential availability of OS and mitochondrial function biomarkers in DR, we conducted a comparative cross-sectional study on different stages of development in DR, DM w/o DR, and healthy volunteers. TAC is progressively diminished the more DR progresses to its proliferative stages. GR and NOS may function as biomarkers to differentiate the progression from S NPDR to PDR, since the behavior of these antioxidant and pro-oxidant biomarkers, respectively, seems to constantly increase neovascularization and deplete its concentration in PDR. CARB acted as early biomarkers where the major difference between groups could be seen when comparing M NPDR to S NPDR, and thus high nmol/mL of CARB may correlate with the progression to S NPDR. H_2_O_2_ was higher in patients with DR compared to DM w/o DR expressing OS in the early development of DR. Regarding mitochondrial function, ATPase is increasingly augmented the more DR progresses, with a statistically significant increase in PDR, functioning as a biomarker that may reflect the difference between N PDR and PDR. ATP synthesis was lower the more DR progressed; however, it was not statistically different between DR groups, but significantly lower compared to DM w/o DR. OS and mitochondrial function biomarkers’ behavior in several stages of DR may better represent the progression and prognosis of DR.

In terms of the limitations of the study, we accept that it is a cross-sectional study with a small sample size. To avoid falling into a type two error, a sample size calculation was provided; however, future studies with larger sample sizes and a follow-up study with multiple determinations in time are required to corroborate these findings and confirm the statistical power of OS and mitochondrial function biomarkers in DR patients.

## Figures and Tables

**Table 1:** Demographic data of patients with DR.

	DM w/o DR *n* = 10	M NPDR *n* = 12	S NPDR *n* = 11	PDR *n* = 9	*p*			
					KW * or X^2 †^	M NPDR vs. S NPDR	M NPDR vs. PDR	S NPDR vs. PDR
Male *n* (%)	5 (50%)	3 (25)	7 (63.63)	8 (88.88)	0.020 **^†^^◊^**	0.083	0.021 **^◊^**	0.366
Female *n* (%)	5 (50%)	9 (75)	4 (36.36)	1 (11.11)				
Age years ± SD	41.2 ± 8.52	60.58 ± 5.35	66.09 ± 9.45	61.33 ± 7.59	<0.001 *^◊^	0.174	0.643	0.238
Smoking history *n* (%)	0 (0%)	4 (33.37)	3 (27.28)	2 (22.22)	0.096 **^†^**	0.933	0.86	0.83
Hypertension *n* (%)	0 (0%)	7 (58.33)	6 (54.54)	8 (88.89)	0.007 **^†◊^**	0.333	0.091	0.763
Dyslipidemia *n* (%)	0 (0%)	3 (25)	1 (9.09)	1 (11.11)	0.091 **^†^**	0.736	0.763	0.564
DM duration years ± SD	7.55 ± 3.80	18.25 ± 4.11	21.45 ± 8.95	18.89 ± 12.70	<0.001 *^◊^	0.174	0.887	0.492
DR duration years ± SD	NA	0.71 ± 1.76	3.00 ± 3.41	2 ± 2.60	<0.001 *^◊^	0.093	0.289	0.488

Values are expressed in means and standard deviations. Abbreviations: M NPDR = Moderate Non-Proliferative Diabetic Retinopathy; S NPDR = Severe Non-Proliferative Diabetic Retinopathy; PDR = Proliferative Diabetic Retinopathy; DM = diabetes mellitus; NA = not applicable; KW * = Kruskal–Wallis test with post hoc Dunn–Bonferroni test; * = Values with “*” have been determined with KW test; X^2 **†**^ = Chi2 test; † = Values with “†” have been determined with X^2^; SD = standard deviation; and BMI = Body Mass Index. ◊ = statistical significance. Mann–Whitney U-test was used to compare differences between DR groups (M NPDR vs. S NPDR, M NPDR vs. PDR, and S NPDR vs. PDR).

**Table 2:** Clinical and ophthalmological parameters.

	DM w/o DR *n* = 10	M NPDR *n* = 12	S NPDR *n* = 11	PDR *n* = 9	*p*			
					KW	M NPDR vs. S NPDR	M NPDR vs. PDR	S NPDR vs. PDR
Weight (kg)	65.7 ± 13.49	69.39 ± 11.41	71.39 ± 8.39	78.04 ± 8.13	0.049 **^◊^**	0.517	0.055	0.128
BMI (kg/m^2^)	22.34 ± 1.94	26.29 ± 2.27	27.23 ± 2.23	27.17 ± 1.97	<0.001 **^◊^**	0.156	0.200	0.676
SBP (mmHg)	118 ± 8.33	129.17 ± 10.03	129.36 ± 4.5	134.22 ± 4.99	<0.001 **^◊^**	0.403	0.116	0.033 **^◊^**
DBP (mmHg)	74 ± 6.50	76.83 ± 4.90	77.82 ± 4.53	80.44 ± 3.81	<0.001 **^◊^**	0.578	0.114	0.420
IOP (mmHg) OD	15.7 ± 2.31	14.17 ± 1.85	14.67 ± 4.03	13.89 ± 1.36	0.208	0.998	0.942	0.964
IOP (mmHg) OS	15.1 ± 1.79	14.5 ± 1.98	14.89 ± 3.52	13.56 ± 1.51	0.054	0.855	0.315	0.445
BCVA (LogMar) OD	0.05 ± 0.11	0.20 ± 0.17	0.27 ± 0.23	0.68 ± 0.44	<0.001 **^◊^**	0.471	0.005 **^◊^**	0.024 **^◊^**
BCVA (LogMar) OS	0.07 ± 0.11	0.16 ± 0.11	0.28 ± 0.21	0.53 ± 0.29	0.006 **^◊^**	0.133	0.006 **^◊^**	0.032 **^◊^**

Values are expressed in means ± standard deviations (SDs). Abbreviations: M NPDR = Moderate Non-Proliferative Diabetic Retinopathy; S NPDR = Severe Non-Proliferative Diabetic Retinopathy; PDR = Proliferative Diabetic Retinopathy; KW = Kruskal–Wallis with post hoc Dunn–Bonferroni test; SD = standard deviation; SBP = Systolic Blood Pressure; DBP = Diastolic Blood Pressure; IOP = intraocular pressure; OD = Right Eye; and OS = Left Eye. ◊ = statistical significance. Mann–Whitney U-test was used to compare differences between DR groups (M NPDR vs. S NPDR, M NPDR vs. PDR, and S NPDR vs. PDR).

**Table 3:** Biochemical parameters of patients with DR.

	DM w/o DR *n* = 10	M NPDR *n* = 12	S NPDR *n* = 11	PDR *n* = 9	*p*			
					KW	M NPDR vs. S NPDR	M NPDR vs. PDR	S NPDR vs. PDR
HbA1c (%)	5.37 ± 0.44	7.86 ± 0.41	8.04 ± 0.65	8.46 ± 0.38	<0.001 **^◊^**	0.205	0.005 **^◊^**	0.136
Glycemia (mg/dL)	92.5 ± 4.74	147.92 ± 37.25	168.55 ± 68.01	123.22 ± 45.07	<0.001 **^◊^**	0.498	0.166	0.138
Cr (mg/dL)	0.78 ± 0.10	0.8 ± 0.13	0.89 ± 0.15	0.96 ± 0.08	0.011 **^◊^**	0.109	0.004 **^◊^**	0.238
eGFR (mL/min/1.73 m^2^)	104.87 ± 12.82	85.86 ± 15.78	78.85 ± 11.62	87.22 ± 12.63	0.002 **^◊^**	0.268	0.434	0.102
Urea (mg/dL)	15.6 ± 6.22	29.63 ± 8.76	38.3 ± 13.05	32.10 ± 14.54	<0.001 **^◊^**	0.062	0.831	0.403
UA (mg/dL)	5.31 ± 0.94	4.1 ± 0.48	4.74 ± 1.21	6.05 ± 1.63	0.003 **^◊^**	0.659	0.022 **^◊^**	0.065
AST (U/L)	29.1 ± 9.71	25.95 ± 4.29	22.01 ± 3.71	23.98 ± 8.16	0.001 **^◊^**	0.036 **^◊^**	0.543	0.282
ALT (U/L)	19.8 ± 6.14	24.81 ± 7.50	20.05 ± 4.16	19.99 ± 5.91	0.004 **^◊^**	0.057	0.186	0.758
TB (mg/dL)	0.88 ± 0.13	0.45 ± 0.10	0.47 ± 0.11	0.48 ± 0.13	<0.001 **^◊^**	0.392	0.616	0.844
TC (mg/dL)	122.1 ± 25.35	159.09 ± 20.02	170.36 ± 18.6	198.22 ± 25.62	<0.001 **^◊^**	0.094	0.001 **^◊^**	0.025
HDL (mg/dL)	65..1 ± 8.26	43.58 ± 11.18	37.01 ± 10.4	34.33 ± 4.36	<0.001 **^◊^**	0.270	0.035 **^◊^**	0.232
LDL (mg/dL)	51.9 ± 12.94	91.88 ± 18.4	98.55 ± 21.77	101.13 ± 10.41	<0.001 **^◊^**	0.468	0.166	0.870
Trig (mg/dL)	90.2 ± 14.45	175.73 ± 46.05	205.11 ± 35.48	217.00 ± 64.65	<0.001 **^◊^**	0.068	0.119	0.930

Values are expressed in means ± standard deviations (SDs). Abbreviations: M NPDR = Moderate Non-Proliferative Diabetic Retinopathy; S NPDR = Severe Non-Proliferative Diabetic Retinopathy; PDR = Proliferative Diabetic Retinopathy; KW = Kruskal–Wallis with post hoc Dunn–Bonferroni test; SD = standard deviation; HbA1c = glycosylated hemoglobin; Cr = Creatinine; eGFR = estimated Glomerular Filtration Rate; UA = Uric Acid; AST = Aspartate transaminase; ALT = Alanine transaminase; TB = Total Bilirubin; TC = Total Cholesterol; HDL = High-Density Lipoprotein; LDL = Low-Density Lipoprotein; and Trig = Triglycerides. ◊ = statistical significance. Mann–Whitney U-test was used to compare differences between DR groups (M NPDR vs. S NPDR, M NPDR vs. PDR, and S NPDR vs. PDR).

**Table 4:** Oxidative stress and mitochondrial function biomarkers.

	DM w/o DR *n* = 10	M NPDR *n* = 12	S NPDR *n* = 11	PDR *n* = 9	*p*			
					KW	M NPDR vs. S NPDR	M NPDR vs. PDR	S NPDR vs. PDR
Oxidative Stress Biomarkers								
TAC (mEq/mL)	20.42 ± 2.46	16.05 ± 4.46	15.18 ± 5.12	10.69 ± 5.95	<0.001 **^◊^**	0.580	0.033 **^◊^**	0.053
GR (U/L)	30.98 ± 3.46	54.95 ± 6.71	59.25 ± 6.76	12.75 ± 4.25	<0.001 **^◊^**	0.142	0.116	0.024 **^◊^**
NOS (pmol/mL)	6.28 ± 1.34	7.57 ± 1.18	7.74 ± 1.62	8.66 ± 0.52	0.002 **^◊^**	0.781	0.102	0.216
CARB (nmol/mL)	2.27 ± 0.42	2.74 ± 0.67	3.85 ± 1.08	4.21 ± 1.07	<0.001 **^◊^**	0.034 **^◊^**	0.208	0.237
H_2_O_2_ (µM/L)	2.13 ± 0.32	3.27 ± 1.09	3.21 ± 0.99	2.93 ± 0.78	<0.001 **^◊^**	0.878	0.425	0.511
Mitochondrial Function Biomarkers								
ATPase (F0/F1-ATPasa)	200.27 ± 14.37	257.50 ± 32.48	261.41 ± 33.80	310.83 ± 28.48	<0.001 **^◊^**	0.469	0.003 **^◊^**	0.002 **^◊^**
ATP synthesis (nmol/min mg protein)	130.13 ± 16.79	110.13 ± 14.38	108.32 ± 22.69	92.91 ± 27.59	0.001 **^◊^**	0.665	0.318	0.198

Values are expressed in mean ± standard deviations (SDs). Abbreviations: M NPDR = Moderate Non-Proliferative Diabetic Retinopathy; S NPDR = Severe Non-Proliferative Diabetic Retinopathy; PDR = Proliferative Diabetic Retinopathy; KW = Kruskal–Wallis with post hoc Dunn–Bonferroni test; TAC = Total Antioxidant Capacity; GR = Glutathione Reductase; NOS = Nitric Oxide Synthase; CARB = carbonylated Proteins; and H_2_O_2_ = hydroperoxides. ◊ = statistical significance. Mann–Whitney U-test was used to compare differences between DR groups (M NPDR vs. S NPDR, M NPDR vs. PDR, and S NPDR vs. PDR).

## Data Availability

The data presented in this study are available on request from the corresponding author, due to privacy reasons.
